# Bioinformatics analysis of prognostic value and immunological role of MeCP2 in pan-cancer

**DOI:** 10.1038/s41598-022-21328-8

**Published:** 2022-11-02

**Authors:** Yanfeng Wang, Yunqing Zhang, Fenghui Wang, Ting Li, Xinqiu Song, Haiyan Shi, Juan Du, Huahua Zhang, Hongmei Jing, Jiaqi Han, Dongdong Tong, Jing Zhang

**Affiliations:** 1 Department of Cell Biology and Genetics, Medical College of Yan’an University, No. 38, Guanghua Road, Yan’an, 716000 Shaanxi Province People’s Republic of China; 2 Clinical Laboratory of Affiliated Hospital of Yan’an University, Yan’an, 716000 Shaanxi Province People’s Republic of China; 3 Laboratory of Obstetrics and Gynecology, Affiliated Hospital of Yan’an University, Yan’an, 716000 Shaanxi Province People’s Republic of China; 4 Department of Cell Biology and Genetics, School of Basic Medical Sciences, Xi’an Jiaotong University Health Science Center, Xi’an, 710061 Shaanxi People’s Republic of China; 5 Department of Anesthesiology, Northwest Women’s and Children’s Hospital, Xi’an, 710061 Shaanxi People’s Republic of China

**Keywords:** Cancer genetics, Cancer prevention, Oncogenes, Tumour biomarkers, Tumour immunology

## Abstract

Methyl-CpG-binding protein 2(MeCP2) is an important epigenetic regulatory factor that promotes many tumor developments, such as liver cancer, breast cancer, and colorectal cancer. So far, no pan-cancer analysis has been reported. Therefore, this study aims to explore pan-cancer's prognostic value, immune infiltration pattern, and biological function. We used bioinformatics methods to analyze the expression and prognostic significance of MeCP2, and the relationship between MeCP2 and clinicopathological parameters, genetic variation, methylation, phosphorylation, immune cell infiltration, and biological function in pan-cancer from using a public database. The results showed that expression of MeCP2 was up-regulated in 8 cancers and down-regulated in 2 cancers, which was remarkably correlated with the prognosis, pathological stage, grade and subtype of cancers. The promoter methylation level of MeCP2 DNA was decreased in bladder urothelial carcinoma (BLCA), breast invasive carcinoma (BRCA), liver hepatocellular carcinoma (LIHC), prostate adenocarcinoma (PRAD), uterine corpus endometrial carcinoma (UCEC), testicular germ cell tumors (TGCT), and stomach adenocarcinoma (STAD);decreased phosphorylation of S25, S90, S92, S241, S286, S325 and S435 was found in MeCP2, such as UCEC, lung adenocarcinoma (LUAD), ovarian serous cystadenocarcinoma (OV), colon adenocarcinoma (COAD), and kidney renal clear cell carcinoma (KIRC). Furthermore, MeCP2 expression was significantly associated with multiple immunomodulators and immune cell infiltration levels across most tumors. Therefore, our pan-cancer explored the prognostic markers and immunotherapeutic value of MeCP2 in different cancers.

## Introduction

Worldwide, the incidence and mortality of cancer are increasing rapidly year by year. According to World Health Organization (WHO) annual report, there are an estimated 19.3 million new cancer cases and nearly 10 million cancer deaths in the world in 2020, which is the leading cause of human death^1^. At present, although the clinical treatment of tumors mainly improves the survival of tumor patients through surgery combined with radiotherapy, chemotherapy, immunotherapy and targeted drug therapy, the cytotoxicity and drug resistance caused by side effects in the treatment process still make the survival rate of patients low^1,2^. Notably, early diagnosis and treatment of tumors can effectively improve the prognosis of patients^3^. Therefore, there is an urgent need for a novel diagnostic marker and therapeutic target molecule for cancer patients.

Methyl-CpG-binding protein 2(MeCP2) is an X-linked gene, belonging to one of the members of methyl CpG binding domain (MBD) family members. The structure contains MBD and transcriptional repression domain (TRD), which can inhibit or promote gene transcription^4,5^. Previous studies found that MeCP2, as a new oncogene, was amplified in cancer cell lines and played a role in tumor cell proliferation, cell cycle, apoptosis, and invasion in several cancer types, such as osteosarcoma and gastric cancer^6–8^. Notably, HDAC inhibitor-mediated MeCP2 can be used in the treatment of tumors. For example, HDAC inhibitor sodium butyrate-treated HeLa cells to induce global genomic histone acetylation, significantly reducing MeCP2^9–11^. Additionally, pan-cancer analysis based on public databases can reasonably identify the common characteristics and heterogeneity of tumors, determine the biological functions of some genes in tumors, and provide new therapeutic targets for patient treatment^12–14^. Therefore, through pan-cancer analysis, it is found that the detection of HER2 can benefit from the precise diagnosis and targeted therapy of head and neck squamous cell carcinoma (HNSC), non-small cell lung cancer (NSCLC), (CESC), BLCA, and UCEC^15^.

**Figure 1:**
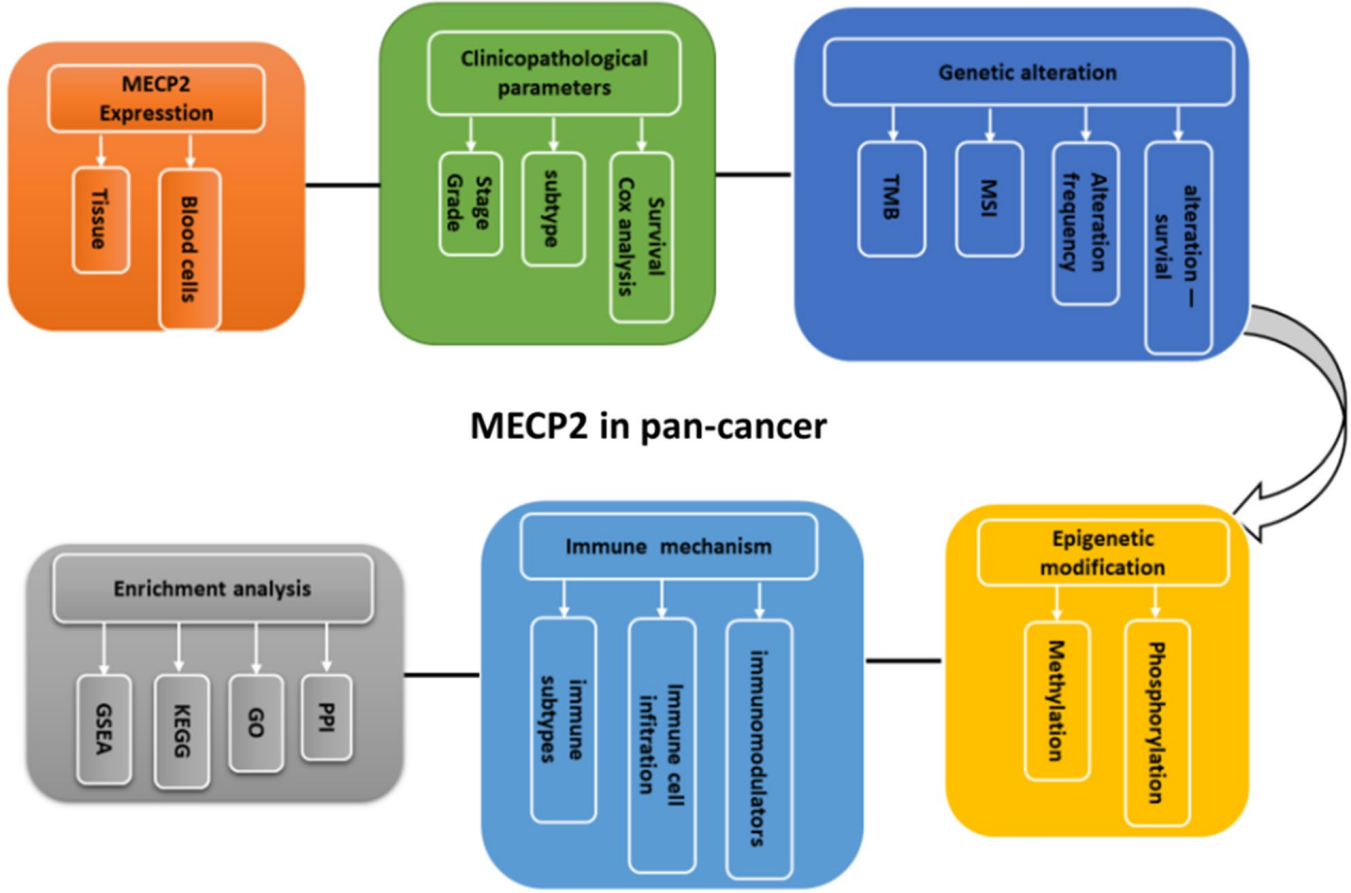
The workflow of the current study. *MSI* microsatellite instability, *TMB* tumor mutational burden, *PPI* protein–protein interaction, *GO* gene ontology, *KEGG* Kyoto Encyclopedia of Genes and Genomes, *GSEA* Gene Set Enrichment Analysis.

In this study, the expression of MeCP2 and its prognostic value were analyzed by bioinformatics. Additionally, the effects of MeCP2 expression on tumor mutation load (TMB) and microsatellite instability (MSI) were analyzed, along with DNA methylation, protein phosphorylation, and immune cell infiltration in human tumors. Protein‑protein interaction (PPI), Gene Ontology (GO), Kyoto Encyclopedia of Genes and Genomes (KEGG) and gene set enrichment analysis (GSEA)analysis explore the biological function of MeCP2 in tumors, as demonstrated by the workflow of this study (Fig. 1). Collectively, the results of this study provide new insights for precise cancer diagnosis and targeted immunotherapy.

## Methods

### MeCP2 expression and prognostic values analysis

The Oncomine database (https://www.oncomine.org/resource/login.html) is an online platform for cancer microarray database and comprehensive data mining^16^, which is used to analyze the expression of MeCP2 in different types of tumor tissues and normal tissues (*P* value is 0.001, fold change is 1.5, and gene ranking of all). The expression of MeCP2 in various immune cells of blood was analyzed by the HPA database (http://www.proteinatlas.org/). The expression level of MeCP2 mRNA in pan-cancer and its correlation with clinicopathological parameters were analyzed using data obtained from the Cancer Genome Atlas (TCGA) (https://xena.ucsc.edu). All databases were accessed in January 2022. The expression, tumor stage, histological grade, and survival analysis are based on “ggplot2”,“survival”,“ggcorrplot”, “coxph”, and “forestplot”packages. A comparison of expression and regression analysis was performed with ANOVA. The survival analysis was evaluated using the Kaplan–Meier method and log-rank test. The COX analysis was used to explore the relationship between the expression of MeCP2 and the prognosis of pan-cancer. The *P* value < 0.05 was considered statistical significance.

### MeCP2 genetic alteration analysis

MeCP2 genetic alteration analysis in pan-cancer was performed using cBioPortal (https://www.cbioportal.org/), a public database for visualizing and analyzing multidimensional cancer genomics data, accessed in January 2022. MeCP2 alterations data was downloaded from the cBioPortal online database to analyze the alterations of MeCP2 in Pan-cancer and its relationship with tumor mutation burden (TMB), Microsatellite Instability (MSI), and prognosis. Spearman's method performed a correlation analysis between cancer gene expression and TMB or MSI. *P* < 0.05 was considered to indicate a statistically significant difference.

### MeCP2 epigenetic modification analysis

The UALCAN database (http://ualcan.pathuab.edu/analysis.html) contains 31 kinds of tumor RNA sequences and clinicopathological parameter data. By analyzing cancer omics data, we can deeply analyze the expression of Pan oncogenes^17^. In this study, the UALCAN database was used to analyze the methylation level and protein phosphorylation level of the MeCP2 promoter region in normal and cancer tissues, accessed in January 2022. The significance of differences was evaluated using a student’s t-test, in which *P* < 0.05 was considered significant (*P < 0.05, **P < 0.01, ***P < 0.001, ****P < 0.005, ns: *P* > 0.05).

### MeCP2 immune mechanism analysis

We used the “pan-cancer”module in the Assistant for Clinical Bioinformatics online tool (https://www.aclbi.com/) to analyze the association between MeCP2 expression and tumor immune infiltration with data from the XCELL database. The TISIDB database (http://cis.hku.hk/TISIDB/index.php) can integrate various heterogeneous data types and can be used to explore the interaction of specific genes in different tumors-immune^18^. The relationship between MeCP2 expression and immunological modulators (immunosuppressants, immunostimulators, and MHC molecules), immune subtype, and molecular subtype were explored through the TISIDB database (*P* < 0.05 as a cut-off value). The Wilcox test performed two-group data, in which *P* values less than 0.05 were considered statistically significant, and all databases were accessed in 2022.

### Functional and pathway of MeCP2 enrichment analysis

The STRING datasets (https://string-db.org/) were applied to assess the functional interaction between proteins and further understand the occurrence or development mechanism of diseases^19^. Subsequently, the PPI network of MeCP2 was constructed via the STRING database (minimum required interaction score: low confidence (0.150), max number of interactors to show: no more than 50 interactors). The GEPIA2 database (http://gepia2.cancer-pku.cn/) was used for similar gene detection of MeCP2. The top 50 similar genes of MeCP2 performed Gene Ontology (GO) and Kyoto Encyclopedia of Genes and Genomes (KEGG) pathway enrichment analysis in DAVID (http://david.abcc.ncifcrf.gov/). According to the threshold of significance set as *P* < 0.05, the top 5 GO domains (BP: biological process; MF: molecular function; CC: cellular component) and 6 KEGG pathways were identified. KEGG pathway enrichment analysis of MeCP2 was performed using GSEA software (iz), and the threshold was set at *P* < 0.05. The threshold was set at *P* < 0.05. A *P* < 0.05and a count > 2 were considered the threshold for significant differences, accessing all databases in 2022.

## Results

### MeCP2 expression in tissue and blood immune cells

**Figure 2:**
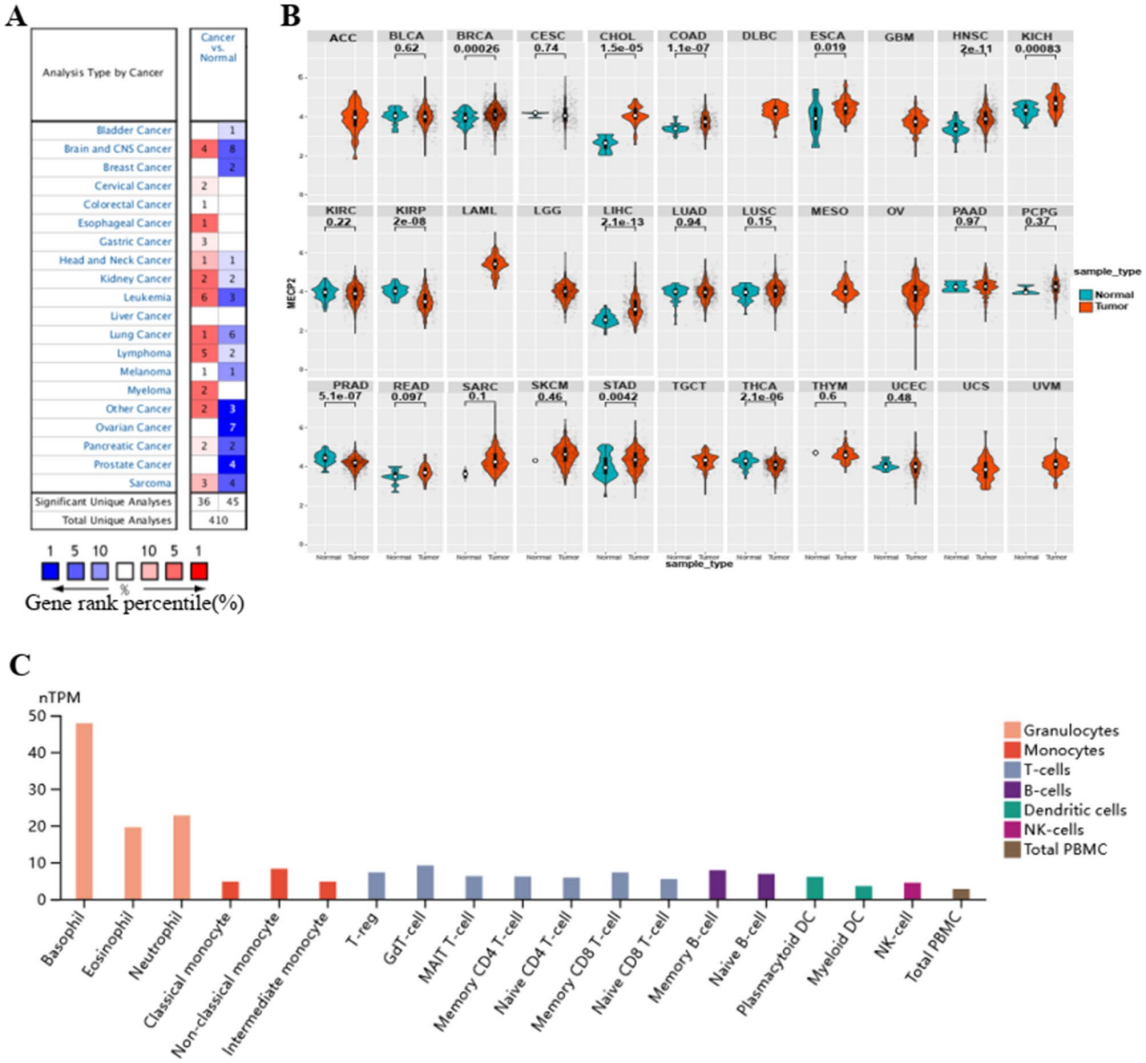
The expression level of the MeCP2 gene in tumors and blood immune cells. **(A)** The expression level of MeCP2 in various cancer tissues compared with normal tissues in the Oncomine database. **(B)** The expression level of MeCP2 in various cancer tissues compared with normal tissues from the TCGA database. **(C)** The expression level of MeCP2 in various blood immune cells was obtained from the HPA dataset.

To explore MeCP2 expression in multiple tumors and normal tissue types, we examined MeCP2 expression via the Oncomine database. The results indicated that MeCP2 expression was higher in the brain, esophageal, kidney, leukemia, lung, and lymphoma cancer, compared to the normal tissues, and lower expression in other different types of cancers, such as brain, breast leukemia, ovarian, and prostate cancers (Fig. 2A). Analysis of MeCP2 mRNA expression level between the tumor and adjacent normal tissues in TCGA database. MeCP2 expression was significantly upregulated in 8 cancers, including BRCA, CHOL, COAD, ESCA, HNSC, KICH, LIHC, and STAD, down-regulated in KIRP, PRAD, and THCA, *P* < 0.05 for all (Fig. 2B). The detail of 33 cancers abbreviations are available in Fig. S1. To further evaluate MeCP2 expression in various blood immune cells, we used the HPA databases. The analysis of immune cell type and lineage specificity showed that MeCP2 expression was enhanced in immune cells (basophils) and lineage-enriched cells (granulocytes; Fig. 2C).

### Correlation analysis between MeCP2 expression and clinicopathological parameters

To better understand the clinical value of MeCP2 expression in pan-cancer, we used the TCGA database to study the relationship between MeCP2 expression and clinicopathological parameters of tumor patients. High expression of MeCP2 was associated with tumor stage in cancers including CHOL, HNSC, KIRC, LIHC, and PAAD (*P* < 0.05) (Fig. S2A) and linked to histological grade in HNSC, KIRC, LGG, and STAD (*P* < 0.05) (Fig. S2B). Additionally, as shown in Fig. S2C, MeCP2 expression has significant correlations with cancer subtypes in most cancers, such as ACC, BRAC, COAD, PCPG, LGG, PRAD, STAD, OV, HNSC, and UCEC (*P* < 0.05).

**Figure 3:**
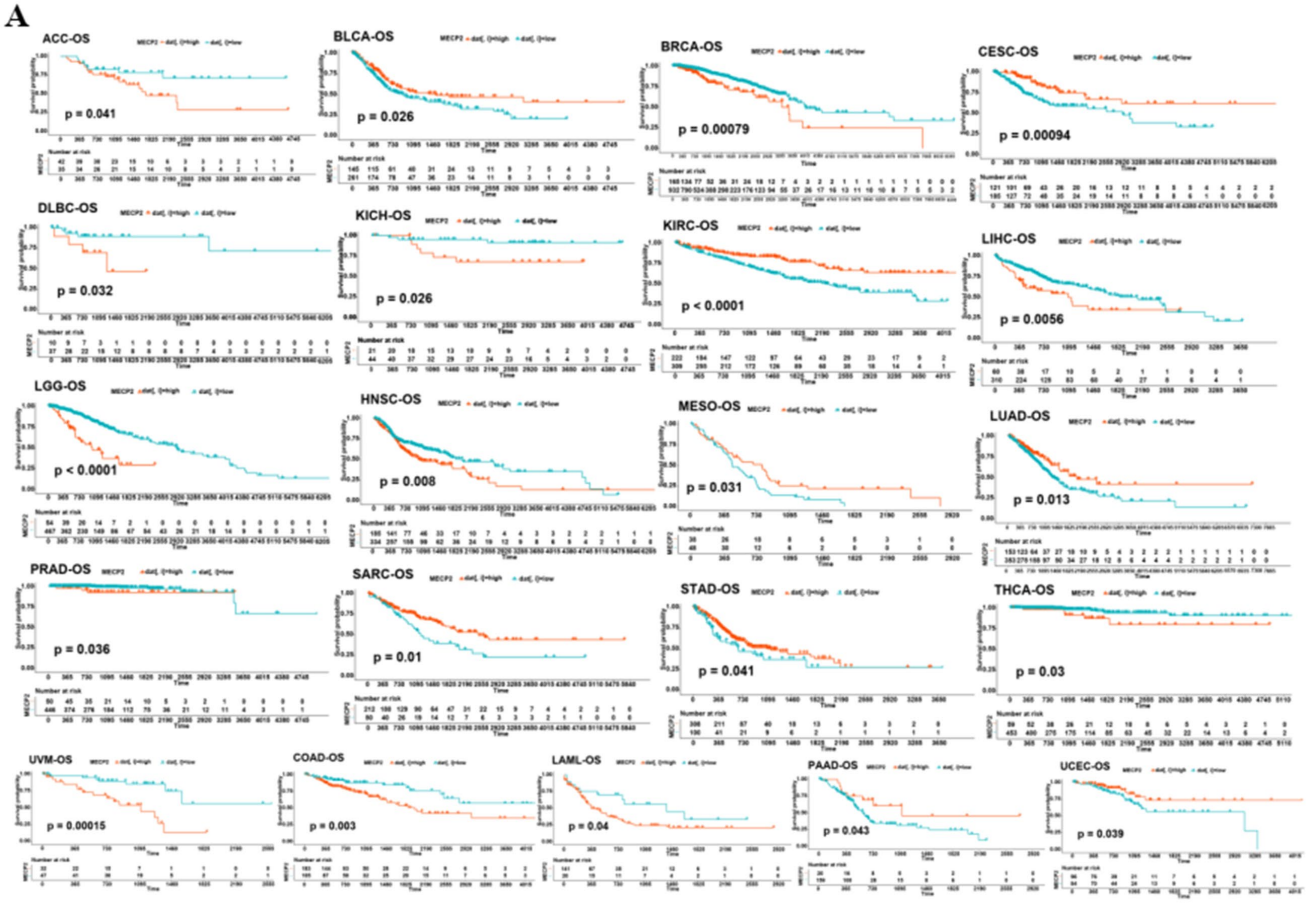

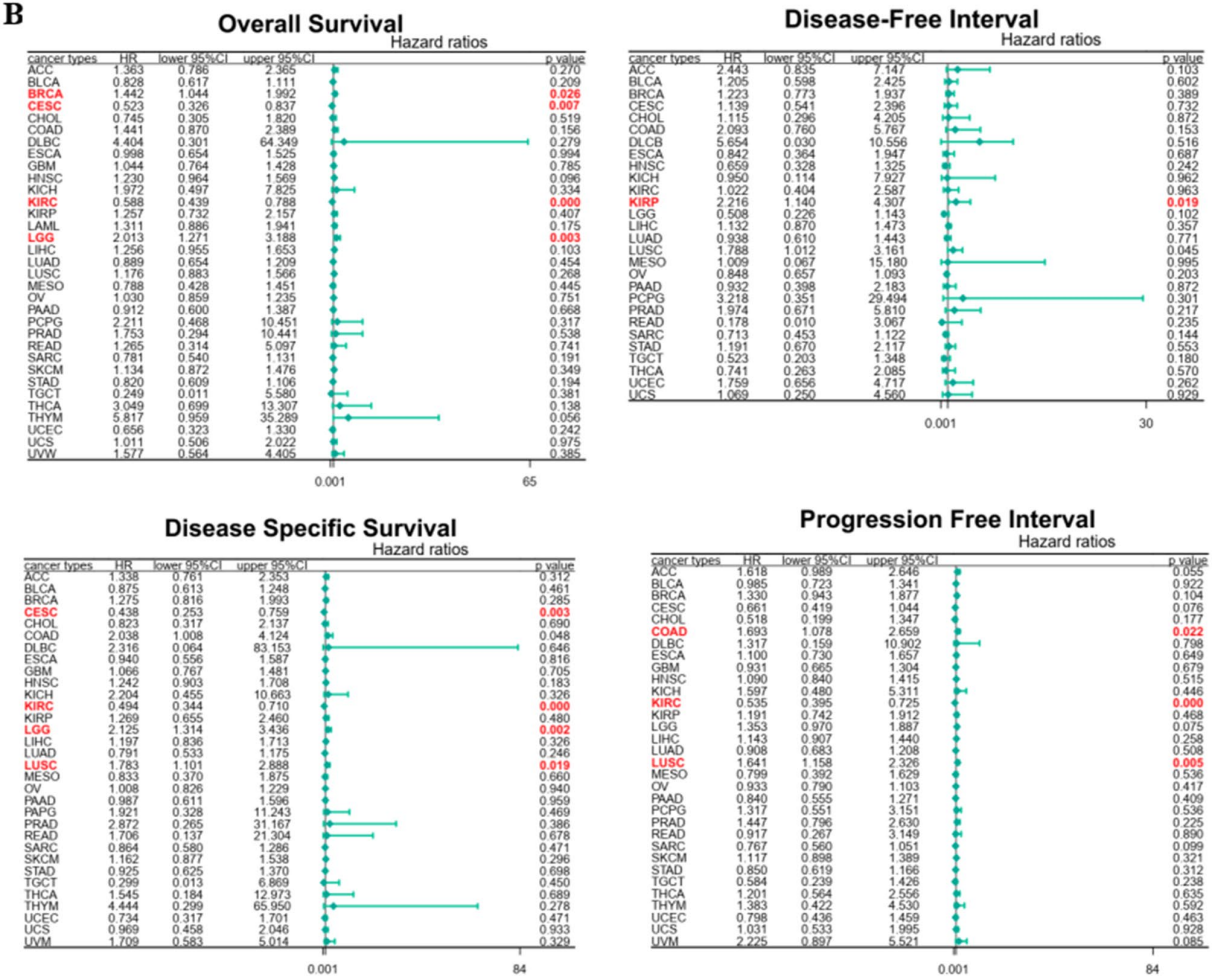
Correlation between MeCP2 gene expression and survival prognosis of pan cancers. **(A)** Survival curves of the relationship between MeCP2 Expression and OS in Pan-cancer. (*P* < 0.05). **(B)** The forest plots of Cox regression analyses MeCP2 Expression and Survival (OS, DFI, PFI, and DSS) (*P* < 0.05). *OS* overall survival, *DFI* disease free interval, *PFI* progression-free interval, *DSS* disease-specific survival, *HR* hazard ratio.

Moreover, the correlation analysis showed that MeCP2 expression had different prognostic values in various types of cancers (Fig. 3). High MeCP2 expression was associated with better overall survival (OS) in certain forms of cancers (BLCA, CESC, KIRC, MESO, LUAD, SARC, STAD, PAAD and UCEC), and worse OS in cancers (ACC, BRCA, DLBC, KICH, LIHC, LGG, HNSC, PRAD, THCA, UVW, COAD, and LAML), *P* < 0.05 for all (Fig. 3A). For disease-specific survival (DSS) outcomes, highly expressed MeCP2 was correlated with a better prognosis of DSS for cancers of BLCA, CESC, KIRC, LUAD, MESO, and UCEC, and was linked with poor prognosis of DSS for cancers of ACC, COAD, HNSC, KICH, LIHC, LUSC, SKCM, THCA, and UVW, *P* < 0.05 for all (Fig. S3A). For progression-free interval (PFI) better outcome, a positive association was apparent between high MeCP2 expression and PFI in CESC, KIRC, STAD, TGCT, and UCEC. In contrast, a negative association was noted in ACC, BRCA, CHOL, LGG, LIHC, LUSC, and UVW, *P* < 0.05 for all (Fig. S3B).

For disease-free interval (DFI) outcome, the better DFI in HNSC, KICH, LGG, OV, SARC, and TGCT were shown to correlate with higher MeCP2 expression, and low MeCP2 expression was related to better DFI in ACC, BRCA, COAD, KIRP, LUSC, and PCPG, *P* < 0.05 for all (Fig. S3C). We also used COX methods to analyze the relationship between MeCP2 and survival (OS, DFI, PFI, and DSS) for further validation. According to the forest plots (Fig. 3B), MeCP2 expression acted as a protective prognostic factor (HR < 1, *P* < 0.05) in CESC (OS), KIRC (OS, DSS, and PFI), and CESC (DSS). MeCP2 expression was considered a risk factor (HR > 1, *P* < 0.05), namely BRCA (OS), LUSC (PFI and DSS), LGG (OS and DSS), and KIRP (DFI and PFI).

### Relationship between MeCP2 expression and genetic alteration

**Figure 4:**
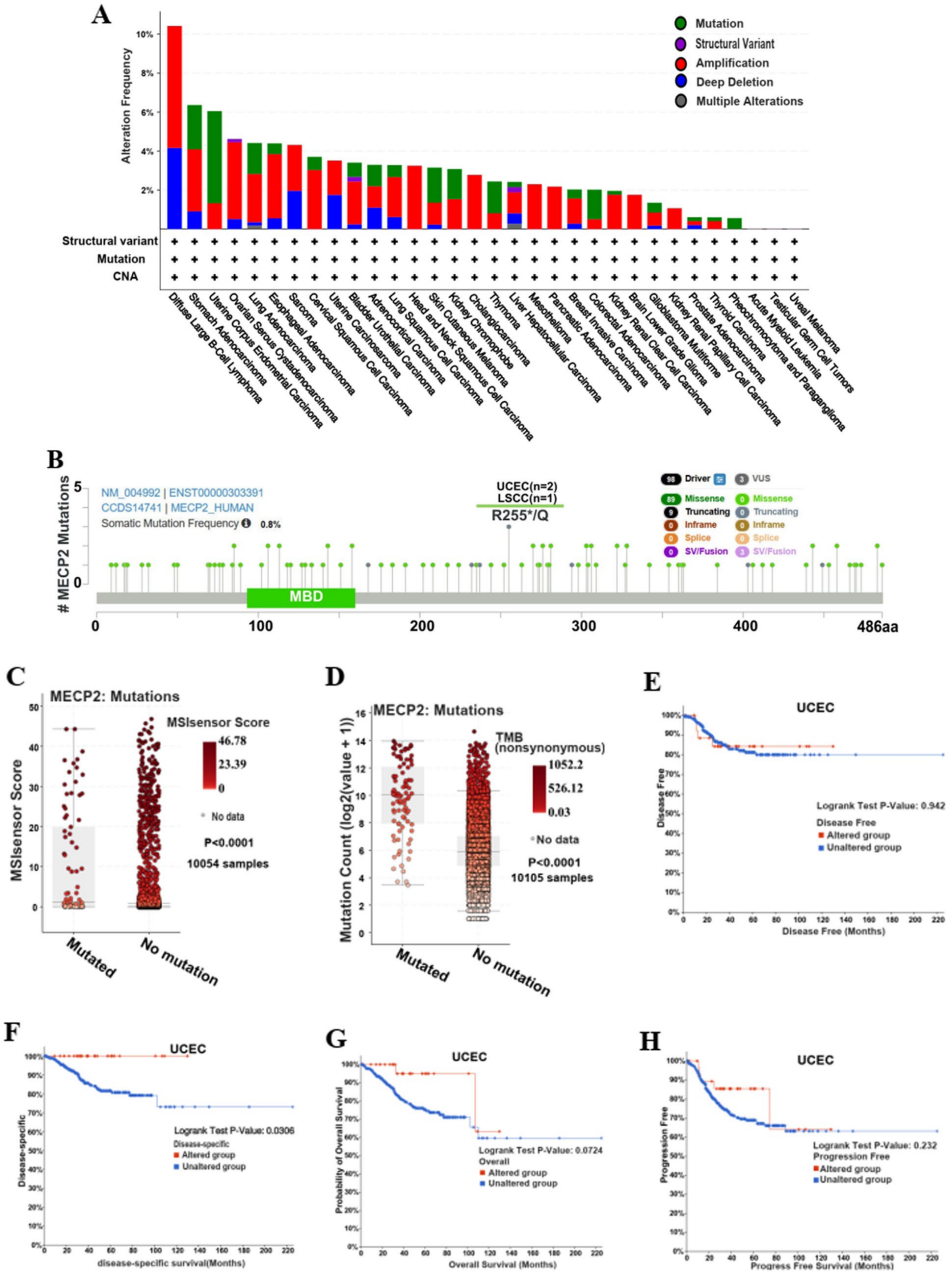

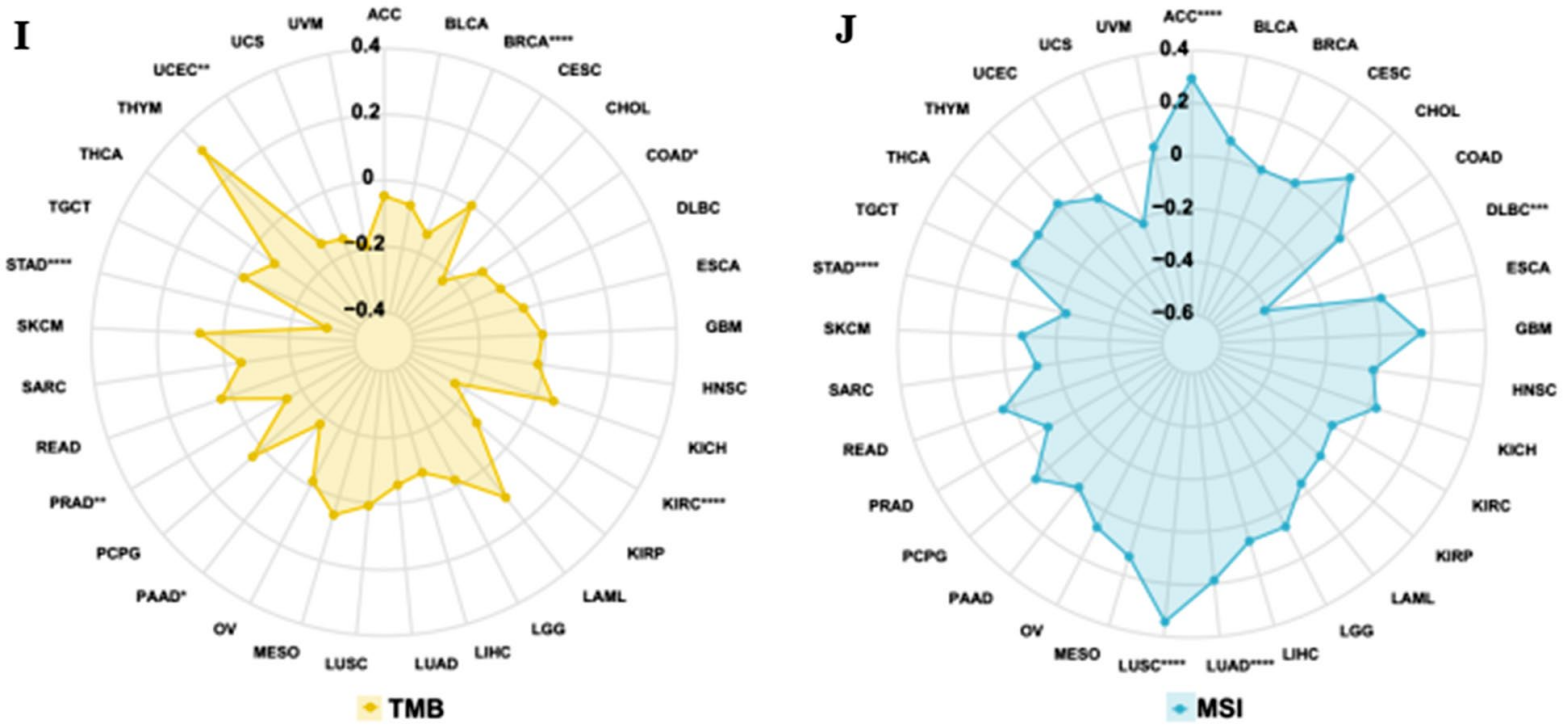
Genetic alterations of MeCP2 in a variety of tumor types. **(A)** The alteration frequency of MeCP2 in different cancers. **(B) **The mutation site of the MeCP2 gene. **(C,D) **The correlation of MeCP2 mutation with MSI sensor score and Mutation count (*P* < 0.0001). **(E,F) **The correlation between MeCP2 mutation and Disease Free, Disease Specific (*P* < 0.05), Overall survival, and Progression Free. **(I–J)** Radar chart displays the correlation between MeCP2 and both TMB andMSI. *P < 0.05, **P < 0.01, ***P < 0.001, ****P < 0.0001. *TMB* tumor mutational burden, *MSI* microsatellite instability.

We investigated the MeCP2 alteration status of Pan cancers from the cBioPortal database. We found that the highest frequency of MeCP2 alteration was associated with amplification, deep deletion, and mutation. As shown in Fig. 4A, diffuse large B-cell lymphoma had the highest frequency of MeCP2 alterations, including amplification and deep deletion (> 10%), followed by stomach adenocarcinoma (> 6%) and uterine corpus endometrial carcinoma (> 6%). The main type of MeCP2 alteration is missense mutations (89), and R255*/Q was detected in UCEC (n = 2) and LSCC (n = 1) (Fig. 4B). Additionally, MeCP2 mutation was significantly correlated with MSI sensor score (10,054 samples) and mutation count (10,105 samples) in the TCGApan-cancer Atlas Studies (*P* < 0.0001) (Fig. 4C,D). The results of Fig. 4E–H showed that altered MeCP2 in UCEC had a better prognosis in disease-specific survival (*P* = 0.0306) than patients in the unaltered group but disease-free (0.942) overall survival (*P* = 0.0724) and progression-free survival (*P* = 0.055) were not different between the two groups. Hence, we investigated the relationship between MeCP2 expression and TMB/MSI in different types of cancer. Our results indicated that MeCP2 expression has significant negative correlations with TMB in STAD, UCEC, BRCA, and KIRC (*P* < 0.05) (Fig. 4I). Finally, we also found that MeCP2 expression has significant negative correlations with MSI in STAD and DLBC while positively correlated with that of ADD, LUAD, and LUSC (*P* < 0.05) (Fig. 4J).

### MeCP2 DNA methylation and protein phosphorylation analysis

**Figure 5:**
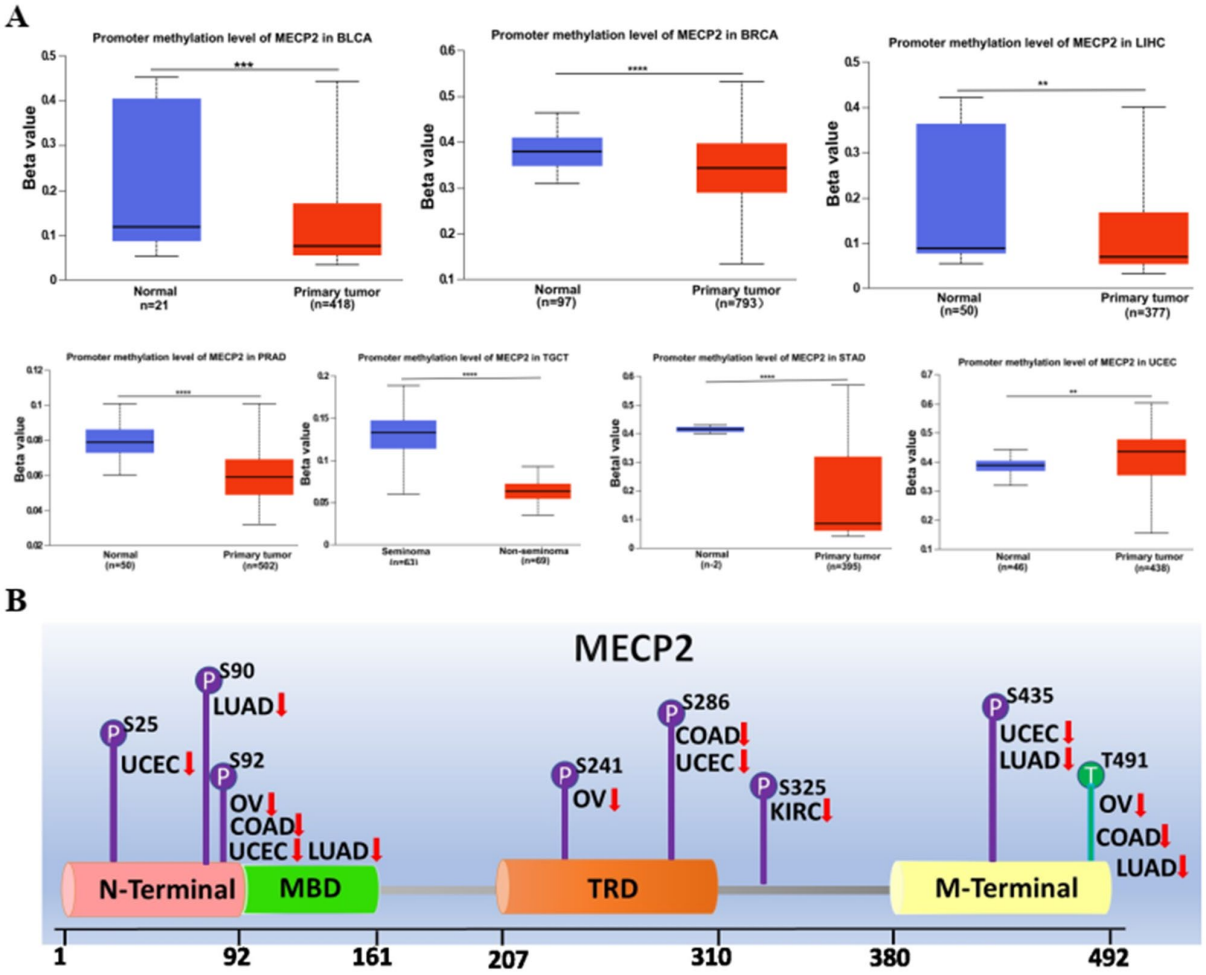

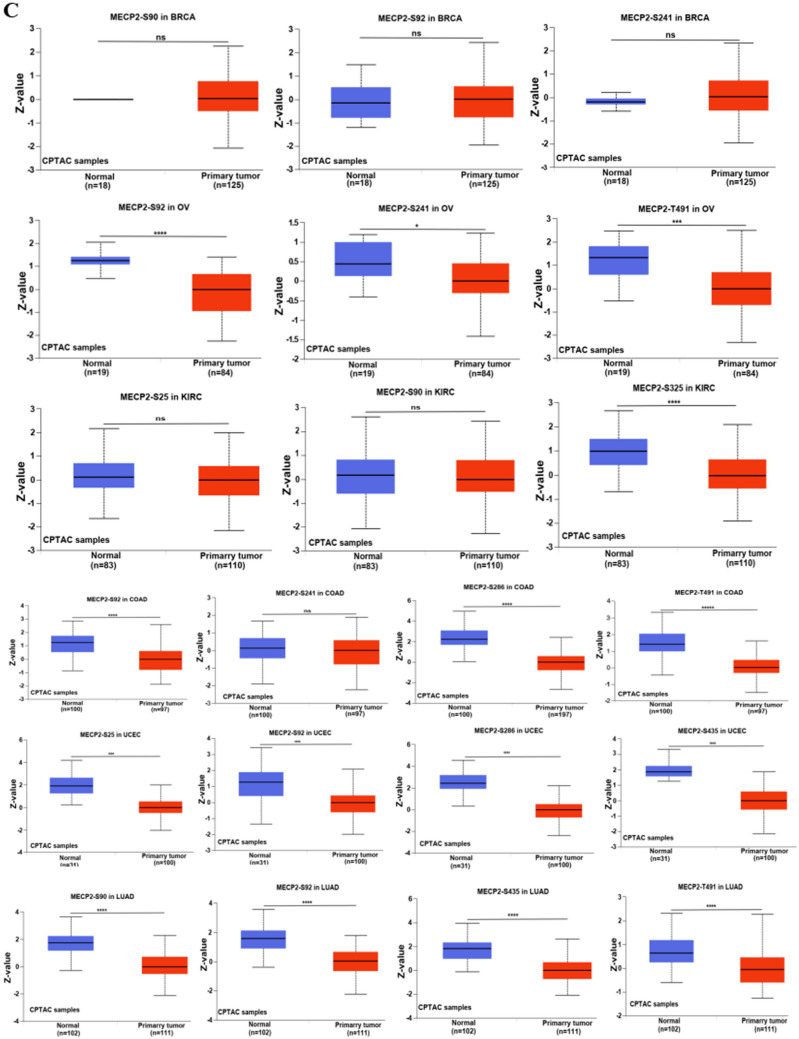
DNA methylation and Phosphorylation analysis of MeCP2protein in several tumors via UALCAN database. **(A)** DNA Promoter methylation level of MeCP2 in BLAC, BRCA, LIHC, PRAD, TGCT and UCEC. **(B)** The schematic diagram of MeCP2 protein phosphoprotein sites. **(C)** The phosphorylation of MeCP2 protein was analyzed in BRCA, OV, KIRC, COAD, UCEC, and LUAD. *P < 0.05, **P < 0.01, ***P < 0.001, ****P < 0.0001, ns:no significance.

DNA methylation and protein phosphorylation directly affect the occurrence and progression of cancer. Using the UALCAN database, we analyzed DNA methylation and protein phosphorylation of MeCP2 in cancers. The results demonstrated that the promoter methylation level of MeCP2 was greatly decreased in BLCA, BRCA, LIHC, PRAD, TGCT, and STAD tissues, while increased in UCEC tissues compared to normal tissues (*P* < 0.05) (Fig. 5A). The schematic diagram of MeCP2 phosphorylation sites is displayed in Fig. 5B. Lower levels of phosphorylation of MeCP2 were observed in OV (S92, S241, and T491), KIRC (S325), COAD (S92, S286, and T491), UCEC (S25, S92, S286, and S435), and LUAD (S90, S92, S435, and T491) compared to normal samples (all *P* < 0.05) (Fig. 5C). The levels of phosphorylation of MeCP2 were no different in BRCA (S90, S92, and S241), and KIRC (S25, S90) (Fig. 5C).

### Association of MeCP2 expression and immune infiltration

**Figure 6:**
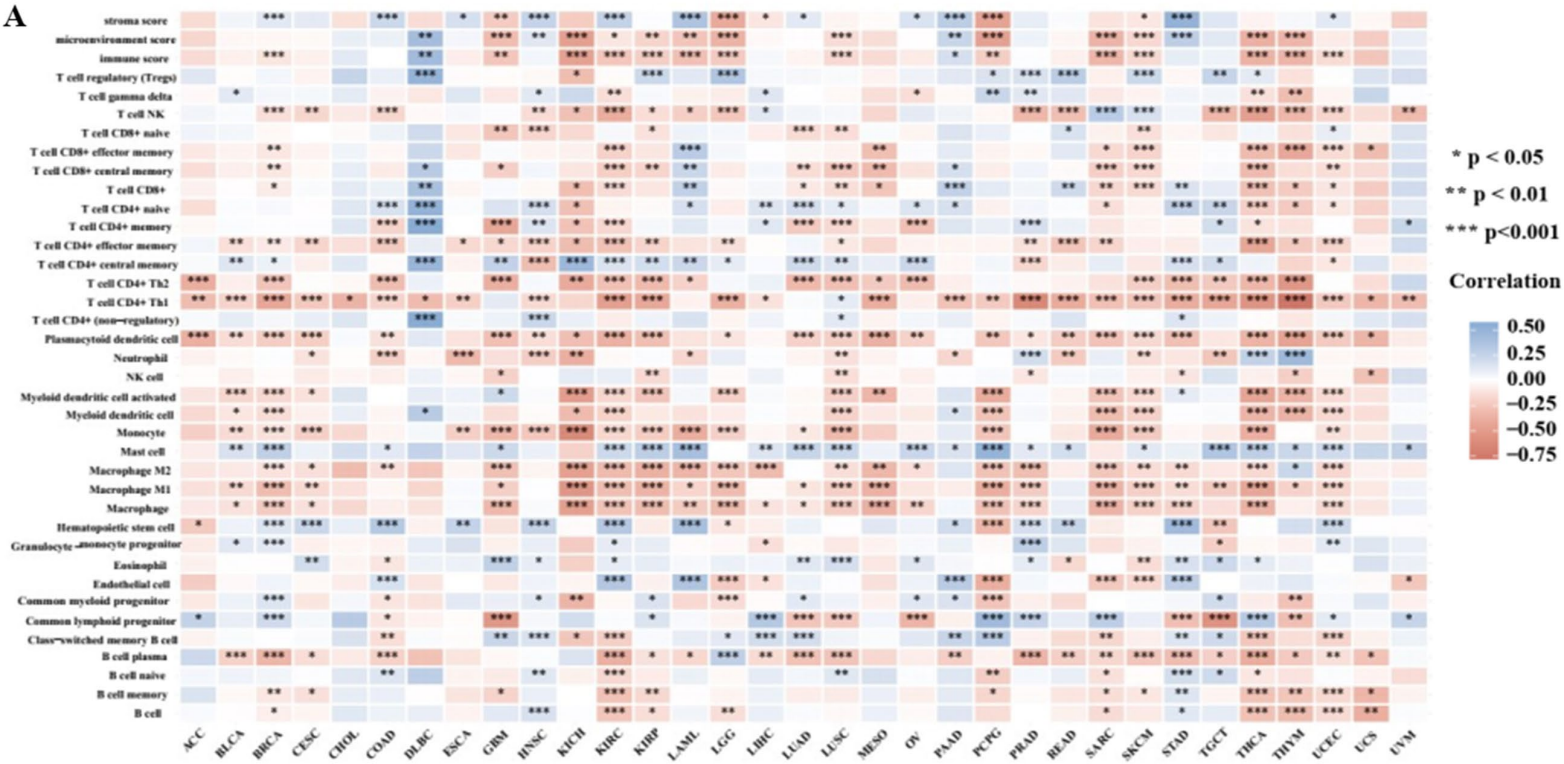

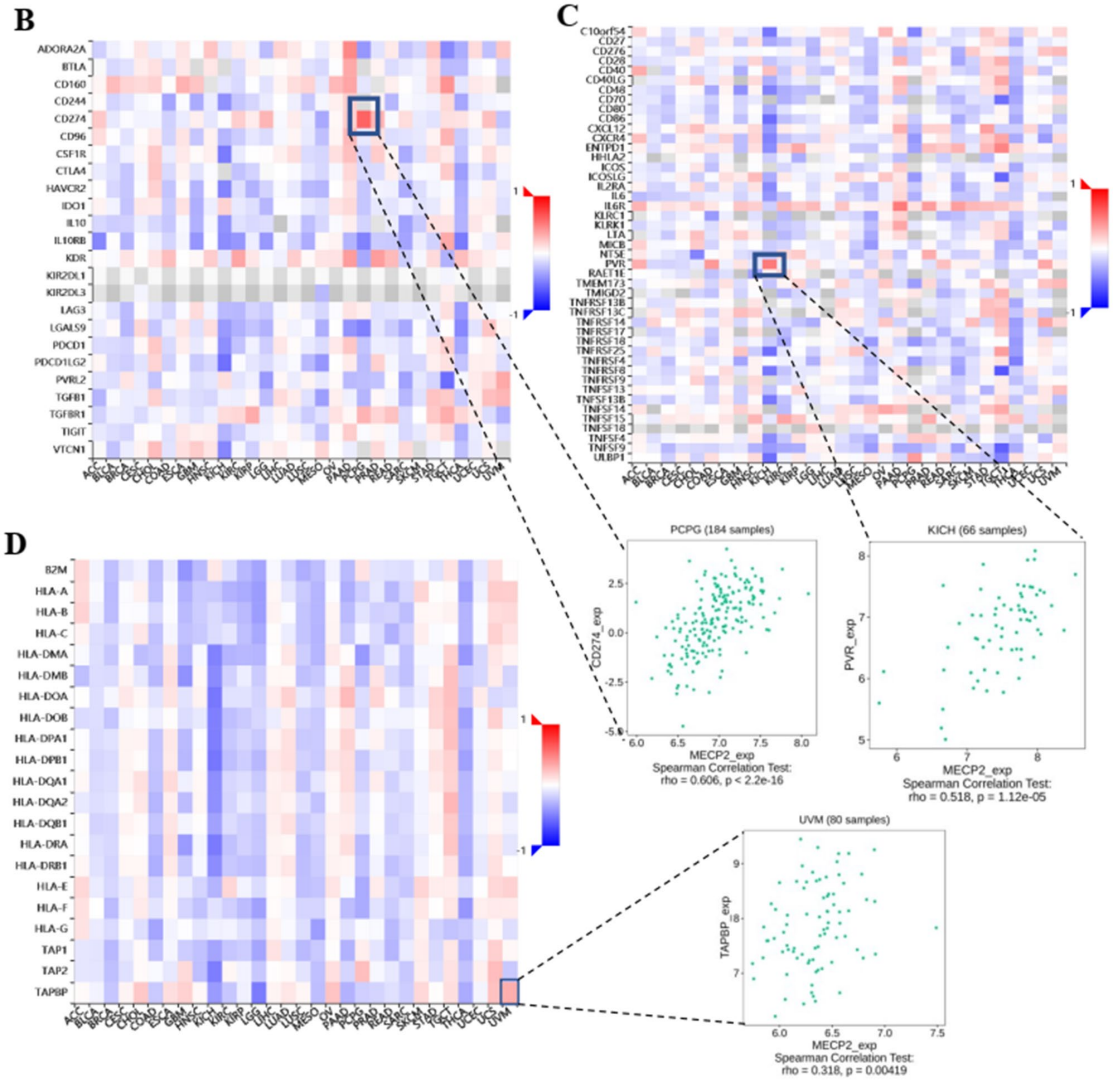
Correlation analysis between MeCP2 expression and immune infiltration. **(A)** The MeCP2 expression correlated with the infiltration levels of various immune cells. **(B)** The MeCP2 expression correlated with the immune inhibitors. **(C)** The MeCP2 expression correlated with the immune stimulators. **(D)** The MeCP2 expression correlated with the MHC molecules. Dot plots displayed the top1 strongest positive correlation. *P < 0.05, **P < 0.01, ***P < 0.001, ****P < 0.0001.

The level of tumor immune infiltration is essential for the survival and immunotherapy of cancer patients. Therefore, we investigated the correlations between MeCP2 expression and immune infiltration levels in 33 cancers using the XCELL database with Assitant for Clinical Bioinformatics online tool. As shown in Fig. 6A, MeCP2 expression was negatively correlated with the stromal score in GBM, LGG, and PCPG and positively correlated with the stromal score in BRCA, COAD, HNSC, KIRC, LAML, PAAD, and STAD (*P* < 0.01). In addition, MeCP2 expression negatively relates to immune scores in BRCA, GBM, KICH, SARC, SKCM, KIRC, KIRP, LAML, LGG, UCEC, LUSC, PCPG, THCA, and THYM, and positively relates to immune score in DLBC and PAAD (*P* < 0.05; Fig. 6A). We also analyzed the correlation between MeCP2 expression and 38 subtypes of immune cells, and the results indicated that MeCP2 expression negatively and significantly correlated with most subtypes in KIRC, KICH, KIRP, LUSC, SARC, SKCM, THCA, THYM, and UCEC, and the expression of MeCP2 were positively and significantly associated with levels of T cell NK, Th1, Th2, M1, M2, B cell plasm, T cell CD4 + central memory, and dendritic cells (all *P* < 0.05; Fig. 6A).

Furthermore, we explored the correlation between MeCP2 expression and three kinds of immunomodulators (immune inhibitor, immunostimulator, and MHC molecule) in cancers via the TISIDB database. Figure 6B shows MeCP2 expression negatively correlated with immune inhibitors in KICH, MESO, and UCEC and positively correlated with immune inhibitors in PCPG (*P* < 0.05). The correlation analyses of immune stimulators indicated that MeCP2 expression was positively associated with PVR in KICH (*P* < 0.05: Fig. 6C). The correlation of MeCP2 expression with MHC molecule was also investigated, UVW, UCS, TGCT, and OV exhibited positive correlations, and KICH, KIRP, LGG, BRCA, and THCAexhibited negative correlation (*P* < 0.05; Fig. 6D). Immune subtypes include wound healing (C1), IFN-γ dominant (C2), inflammatory (C3), lymphocyte depletion (C4), immunologically quiet (C5), and TGF-β dominant (C6). We also found a significant correlation between MeCP2 expression and immune subtypes in most cancers, such as BLCA, BRCA, COAD, HNSC, LGG, LIHC, LUAD, LUSC, KIRC, KIRP, OV, PAAD, PRAD, SARC, PCPG, THCA, STAD and UCEC (*P* < 0.05; Fig. S4).

### PPI, GO, KEGG and GSEA enrichmen analysis of MeCP2-related gene

**Figure 7:**
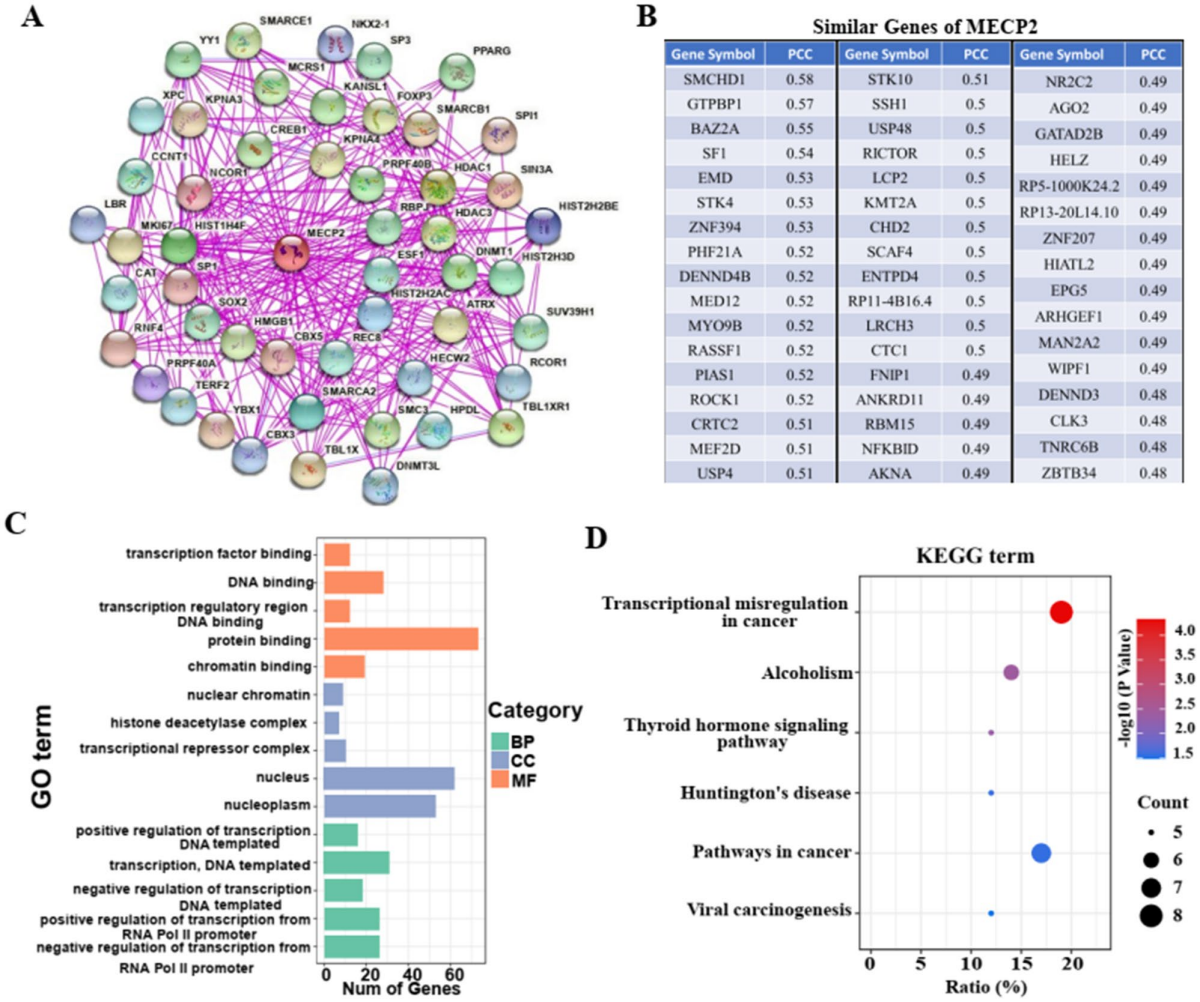
Enrichment analysis of MeCP2-related genes. **(A)** PPI network analysis of MeCP-binding proteins based on STRING database. **(B)** The top 50 similar genes of MeCP2 using the GEPIA2 online tool. **(C)** GO enrichment analysis of MeCP2-related genes. **(D)** KEGG pathway enrichment analysis of MeCP2-related genes. *PPI* protein–protein interaction, *PCC* Pearson correlation coefficient, *GO* Gene Ontology, *BP* biological process, *CC* cell component, *MF* molecular function, *KEGG* Kyoto Encyclopedia of Genes and Genomes.

Considering the robust correlation between the expression of MeCP2 and the analysis results of immune infiltration, TMB, and MSI in STAD, related functional enrichment analyses of MeCP2 were performed via PPI, GO, KEGG and GSEA. Using the STRING online tool, we obtained 50 MeCP2-binding proteins (PPI enrichment *p*-value < 0.05), which have been verified by experiments (Fig. 7A). The top 50 similar genes of MeCP2 were obtained from the GEPIA2 database (all PCC > 0.4:Fig. 7B). By combining the similar genes of MeCP2 and MeCP2-binding proteins, GO, and KEGG pathway enrichment analysis were performed using the DAVID database. The top 15 GO terms are displayed in Fig. 7C. The results indicated that MeCP2-related genes mainly correlated with the biological processes (BP), cellular component (CC), and molecular function (MF), such as transcription factor binding, DNA binding, protein binding, transcriptional repressor complex, positive or negative regulation of transcription DNA templated, and positive or negative regulation of transcription from RNA polymerase II promote (*P* < 0.05). KEGG signaling pathway analysis results revealed that most of these genes were mainly enriched in transcriptional misregulation in cancer, alcoholism, thyroid hormone signaling pathways, Huntington's disease, pathways in cancer, and viral carcinogenesis (*P* < 0.05; Fig. 7D).

To further explore the complex functions of MeCP2 in STAD, the MeCP2 mRNA data of TCGA were analyzed using GSEA analysis of KEGG pathways. The KEGG enrichment analysis results indicated that the high MeCP2 group demonstrated significant positive enrichment in KEGG_BASAL_CELL_CARCINOMA, KEGG_MTOR_SIGNALING_PATHWAY, WNT_SIGNALING_PATHWAY, KEGG_MAPK_SIGNALING_PATHWAY, KEGG_HEDGEHOG_SIGNALING_PATHWAY, and KEGG_PATHWAYS_IN_CANCER (all *P* < 0.05;Fig. S5A). In contrast, the low MeCP2 group demonstrated significant enrichment in KEGG_PROTEASOME,

KEGG_AMINOACYL_TRNA_BIOSYNTHESIS, and KEGG_SPLICEOSOME (all *P* < 0.05;Fig. S5B).

## Discussion

Pan-cancer research can cross the boundaries between tumor types and provide new intervention strategies for clinical trials. Notably, pscreening pan-cancer biomarkers in blood or other body fluids has become a useful, routine, and economic screening tool for better understanding the role of these biomarkers^12,20^. Until recently, the function of MeCP2 in tumor progression has not been extensively studied nor well understood. Although not clearly understood, many studies have shown that MeCP2 can promote tumor progression and metastasis,such as breast cancer,liver cancer,and colorectal cancer^5,21,22^. To better understand the mechanism of MeCP2 in different tumors,we used a series of bioinformatics methods to comprehensively analyze the molecular features of gene expression,genetic alteration,epigenetic modification,and the relationship between MeCP2 expression and survival,TMB,MSI,and immune infiltration in 33 tumor types.

In this study,we first investigated the level of MeCP2 in tissues and blood, combing Oncomine,TCGA,and HPA databases MeCP2 was significantly upregulated in BRCA,CHOL,COAD,E SCA, HNSC, KICH, LIHC, and STAD, but low expression in KIRP, PRAD, and THCA compared with normal tissues (Fig. 2B). In blood immune cells, the MeCP2 lineage is enriched in granulocytes and highly expressed in basophils (Fig. 2C). Interestingly, increased MeCP2 expression is associated with tumor stage, histological grade, and tumor subtype in STAD, HNSC, KIRC, and LGG (Fig. S2). Similar results have also indicated that high MeCP2 expression can promote the proliferation and metastasis of tumor cells, such as gastric cancer, colorectal cancer, and renal cell carcinoma^8,22–24^. Additionally, MeCP2 expression was positively and significantly correlated with the TNM stages, subtypes, and histological types of gastric and breast cancer^25,26^. Furthermore, we found that high MeCP2 expression was correlated with a worse prognosis of OS, DSS, PFI, and DFI in ACC, BRCA, COAD, LIHC, and UVW (Fig. 3A and Fig. S3), and MeCP2 expression served as a prognostic factor in some types of cancer, based on Cox analyses (Fig. 3B). Based on the above results, MeCP2 may be a potential prognostic biomarker for several cancer types and provide a new targeted treatment strategy for various tumor treatments.

Somatic mutations can cause developmental disorders, and the gradual accumulation of mutations in the body can induce cancer^27^. Specific gene mutations are important biomarkers of patient prognosis, which is beneficial in evaluating the effect of drug therapy in cancer patients^28,29^. MeCP2 has a high mutation frequency in diffuse large B-cell lymphomas, such as amplification and deep deletion (Fig. 4A). The main mutation site is R255*/Q in UCEC, and altered MeCP2 is linked to MSI sensor score, mutation count, and survival of disease-specific survival (Fig. 4B–H). It is reported that TMB is a biomarker of the pan-cancer genome, and the level of TMB is related to the production of immunogenic peptides and the efficacy of immune checkpoint inhibitors (ICIs). Additionally, a high level of TMB helps to improve the efficacy of ICIs and OS^30–33^. Our study found that MeCP2 expression was related to TMB of STAD, UCEC, BRCA, and KIRC and linked to MSI of STAD, DLBC, ADD, LUAD, and LUSC (Fig. 4I–J). However, TMB and MSI levels were associated with new antibody production, and TMB levels were elevated in patients with high microsatellite instability (MSI-H)^13,34^, findings consistent with previous experimental results. TMB is an important independent biomarker in MSI-H mCRC, which is used to stratify the possibility of patients' response to ICPIs and plays an essential role in patients' immunotherapy^35^. The high expression of MeCP2 in tumors is related to MSI and TMB, indicating that MeCP2 may be a valuable prognostic biomarker that will benefit immunotherapy and prolonged survival.

Epigenetics can regulate the occurrence and proliferation of cancer, and epigenetic modification is gradually regarded as the key target of tumor research that can better discover the mechanism of tumorigenesis and provide biomarkers for early detection, diagnosis, and treatment of tumor patients^36^. DNA methylation is a critical factor in regulating gene expression, and abnormal DNA methylation can lead to the occurrence and proliferation of cancer^37^. Low DNA methylation levels are remarkably linked to the high MeCP2 expression in BRCA, LIHC, and STAD (Fig. 5A). Previous studies have shown that DNA methylation is a well-characterized epigenetic hallmark of tumors, which can improve the efficacy of chemotherapy for gastric cancer and hepatocellular carcinoma^38,39^. In addition, protein phosphorylation is one of the main post-translational modifications (PTM), including almost all life processes, such as cell division, decomposition, signal transduction, gene expression regulation, and protein interaction. Consequently, mutations in protein phosphorylation sites can lead to tumorigenesis^40,41^. Interestingly, our study showed that the protein phosphorylation level of MeCP2 was decreased in most common cancers, the S241and S286 locus within the TRD in OV, COAD, and UCEC (Fig. 5B,C). A previous study found that the decreased level of phosphorylated PDK-S241 can regulate the protein phosphorylation of the cell cycle and affect the proliferation of prostate cancer^42^. Therefore, the relationship between MeCP2 expression, DNA methylation, and protein phosphorylation needs to be further verified by experiments in tumorigenesis.

To further study the potential value of MeCP2 in pan cancers, we explored the correlation between MeCP2 expression and immune cell infiltration and immunomodulators. MeCP2 expression was correlated with stromal score and immune score in most cancers, and it was significantly associated with levels of immune cells, such as T cell NK, Th1, M1, T cell CD4 + central memory, and dendritic cells (Fig. 6A). Similar results have reported the expression of PD-L1 can promote the strong immune response of tumor-infiltrating immune cells and is significantly correlated with the overall survival in ovarian cancer^43^. In most immune inhibitors, immunostimulators, and MHC molecules, MeCP2 expression was positively associated with CD274(PD-L1), PVR, and TAPBP (Fig. 6C). With the breakthrough of tumor-related immune inhibitors, immunostimulators, and MHC molecules, ICIs have been widely used in tumor immunotherapy and achieved remarkable results. Meanwhile, PD-1/PD-L1 ICIS has been approved as a drug for treating various malignant tumors, including melanoma, lymphoma, lung cancer, HNSC, KIRC, and LIHC^30,44^. Therefore, it is reasonable to speculate that MeCP2 expression may regulate the infiltration level of tumor immune cells and immune responses and ultimately affect the prognosis of tumor patients.

Additionally,100 MeCP2-related genes were performed for functional enrichment analysis in STAD. For the GO terms, MeCP2-related genes are mainly enriched in transcription factor binding, DNA binding, protein binding, regulation of transcription DNA templated, and regulation of transcription from RNA polymerase II (Fig. 7C). MeCP2 dysfunction leads to changes in the expression level of thousands of genes, about 15% inhibited and 85% activated. For example, MeCP2 recruitment of CREB1 can activate the transcription of downstream genes^45^. KEGG pathway enrichment analysis showed that MeCP2 was mainly enriched in pathways related to tumorigenesis, such as transcriptional misregulation in cancer and pathways in cancer. Interestingly, the GSEA results also showed that MeCP2 was significantly related to the signal pathway of tumorigeneses, such as KEGG_PATHWAYS_IN_CANCE, KEGG_BASAL_CELL_CARCINOMA, and WNT_SIGNALING_PATHWAY. Emerging evidence demonstrates that PI3K/AKT/mTOR signaling pathway is significantly correlated with the occurrence, prognosis, and metastasis of gastric cancer, and the PI3K/AKT/mTOR signaling pathway may be a molecular target and potential therapeutic strategy for the clinical diagnosis of gastric cancer^46^. However, further studies are needed to determine the mechanism of MeCP2 and these signaling pathways and to explore whether it can be used as an indicator of tumor diagnosis and prognosis.

## Conclusions

In summary, the results of this study show that MeCP2 expression is significantly related to pathological stage, tumor grade, clinical prognosis, DNA methylation, protein phosphorylation, and immune cell infiltration in CHOL, HNSC, KIRC, LIHC, and STAD. In addition, MeCP2 expression is related to the status of immunotherapy-associated signatures. Therefore, MeCP2 can be used as a prognostic biomarker of pan-cancer and provide a new strategy for treating tumor ICIs.

## Supplementary Information

Supplementary Information.

## Data Availability

The datasets used and/or analysed during the current study available from the corresponding author on reasonable request.
