# Supplementary material for: Bioinformatics analysis of prognostic value and immunological role of MeCP2 in pan-cancer
Source: Sci Rep. 2022 Nov 2;12:18518. doi: 10.1038/s41598-022-21328-8 (PMC9630441; doi:10.1038/s41598-022-21328-8)
Supplement: Supplementary file 1 — Supplementary Information. [file 41598_2022_21328_MOESM1_ESM.pdf]

SUPPLEMENTARY

Bioinformatics analysis of prognostic value and immunological role of MeCP2 in pan-cancer

Yanfeng Wang[1,2,†], Yunqing Zhang[3,†], Fenghui Wang[1,†], Ting Li[5], Xinqiu Song[1], Haiyan Shi[1], Juan Du[1], Huahua Zhang[1], Hongmei Jing[1], Jiaqi Han[1], Dongdong Tong[4,*] and Jing Zhang[1,*]

†These authors contributed equally to this work.

1.Department of Cell Biology and Genetics,Medical College of Yan'an University, Yan'an 716000,Shaanxi Province, China.

2.Clinical Laboratory of Affiliated Hospital of Yan 'an University,Yan'an 716000, Shaanxi Province, China.

3.Laboratory of Obstetrics and Gynecology,Affiliated Hospital of Yan 'an University, Yan'an 716000, Shaanxi Province, China.

4.Department of Cell Biology and Genetics, School of Basic Medical Sciences, Xi'an Jiaotong University Health Science Center, Xi'an 710061,Shaanxi,China.

5.Department of Anesthesiology, Northwest Women's and Children's Hospital, Xi'an, Shaanxi,710061,China.

*Correspondence:

Jing Zhang

Department of Cell Biology and Genetics, Medical College of Yan'an University, No. 38, Guanghua road, Yan'an city, Shaanxi Province,716000, People's Republic of China.

Tel/Fax:+86-0911-2650158

Email address: yadxzj@163.com

Dongdong Tong
Department of Cell Biology and Genetics, School of Basic Medical Sciences, Xi'an Jiaotong University Health Science Center, Shaanxi, Xi'an 710061,People's Republic of China.E-mail address: tongdd@xjtu.edu.cn

SUPPLEMENTARY MATERIAL

S1

Abbreviations	Tumor full name	Abbreviations	Tumor full name
ACC	Adrenocortical carcinoma	LUAD	Lung adenocarcinoma
BLCA	Bladder urothelial carcinoma	LUSC	Lung squamous cell carcinoma
BRCA	Breast invasive carcinoma	MESO	Mesothelioma
CESC	Cervical squamous cell carcinoma and endocervical adenocarcinoma	OV	Ovarian serous cystadenocarcinoma
CHOL	Cholangiocarcinoma	PAAD	Pancreatic adenocarcinoma
COAD	Colon adenocarcinoma	PCPG	PCPG Pheochromocytoma and paraganglioma
DLBC	Lymphoid neoplasm diffuse large B-cell lymphoma	PRAD	Prostate adenocarcinoma
ESCA	Esophageal carcinoma	READ	Rectum adenocarcinoma
GBM	Glioblastoma multiforme	SARC	Sarcoma
HNSC	Head and neck squamous cell carcinoma	SKCM	Skin cutaneous melanoma
KICH	Kidney chromophobe	STAD	Stomach adenocarcinoma
KIRC	Kidney renal clear cell carcinoma	TGCT	Testicular germ cell tumors
KIRP	Kidney renal papillary cell carcinoma	THCA	Thyroid carcinoma
LAML	Acute myeloid leukemia	THYM	Thymoma
LGG	Brain lower grade glioma	UCEC	Uterine corpus endometrial carcinoma
LIHC	Liver hepatocellular carcinoma	UCS	Uterine carcinosarcoma
		UVM	Uveal melanoma

Figure S1. The detail of 33 cancers abbreviations.

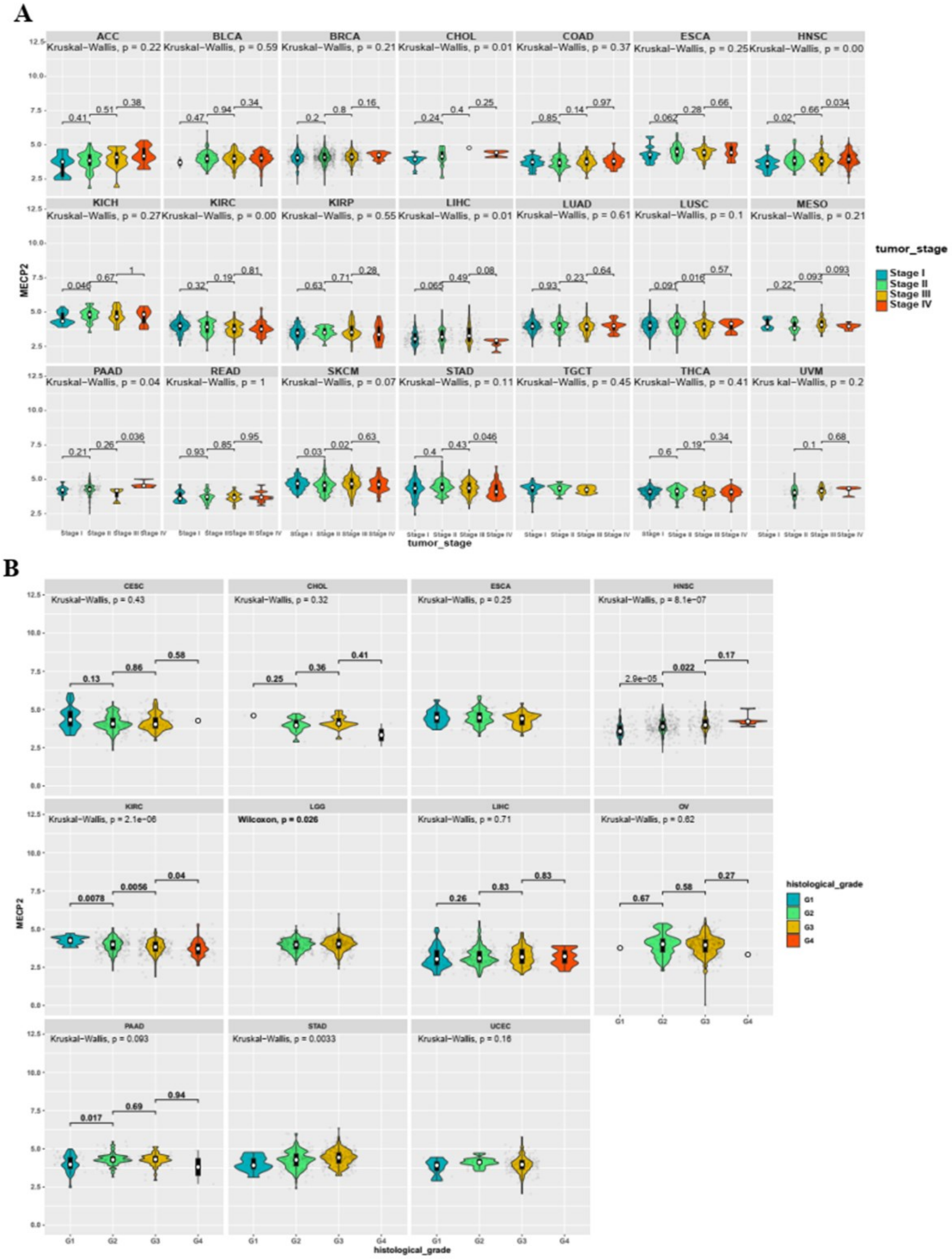
A
ACC
Kruskal-Wallis, p = 0.22
BLCA
Kruskal-Wallis, p = 0.59
BRCA
Kruskal-Wallis, p = 0.21
CHOL
Kruskal-Wallis, p = 0.01
COAD
Kruskal-Wallis, p = 0.37
ESCA
Kruskal-Wallis, p = 0.25
HNSC
Kruskal-Wallis, p = 0.00
KICH
Kruskal-Wallis, p = 0.27
KIRC
Kruskal-Wallis, p = 0.00
KIRP
Kruskal-Wallis, p = 0.55
LIHC
Kruskal-Wallis, p = 0.01
LUAD
Kruskal-Wallis, p = 0.61
LUSC
Kruskal-Wallis, p = 0.1
MESO
Kruskal-Wallis, p = 0.21
PAAD
Kruskal-Wallis, p = 0.04
READ
Kruskal-Wallis, p = 1
SKCM
Kruskal-Wallis, p = 0.07
STAD
Kruskal-Wallis, p = 0.11
TGCT
Kruskal-Wallis, p = 0.45
THCA
Kruskal-Wallis, p = 0.41
UVM
Krus kal-Wallis, p = 0.2
MECP2
tumor_stage
Stage I
Stage II
Stage III
Stage IV
B
CESC
Kruskal-Wallis, p = 0.43
CHOL
Kruskal-Wallis, p = 0.32
ESCA
Kruskal-Wallis, p = 0.25
HNSC
Kruskal-Wallis, p = 8.1e-07
KIRC
Kruskal-Wallis, p = 2.1e-06
LGG
Wilcoxon, p = 0.026
LIHC
Kruskal-Wallis, p = 0.71
OV
Kruskal-Wallis, p = 0.62
PAAD
Kruskal-Wallis, p = 0.093
STAD
Kruskal-Wallis, p = 0.0033
UCEC
Kruskal-Wallis, p = 0.16
MECP2
histological_grade
G1
G2
G3
G4

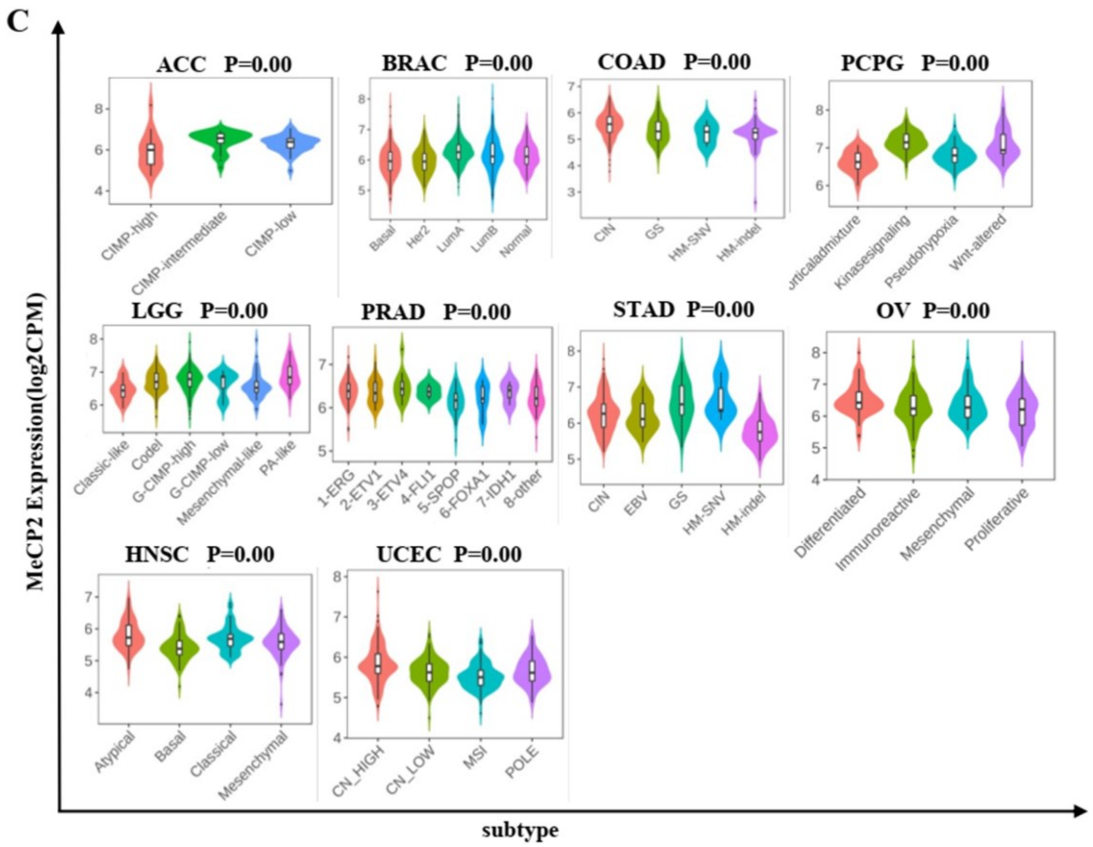

Figure S2. The MeCP2 expression correlated with stage,grade and subtype in pan-cancer.**(A)** High MeCP2 expression correlated with tumor stage in CHOL,HNSC,KIRC,LIHC and PAAD(P<0.05).**(B)** High MeCP2 expression correlated with histological grade in HNSC,KIRC,LGG and STAD(P<0.05).**(C)** High MeCP2 expression in different tumor subtypes in ACC,BRAC,COAD, PCPG,LGG,PRAD,STAD,OV,HNSC and UCEC(P<0.05).

A

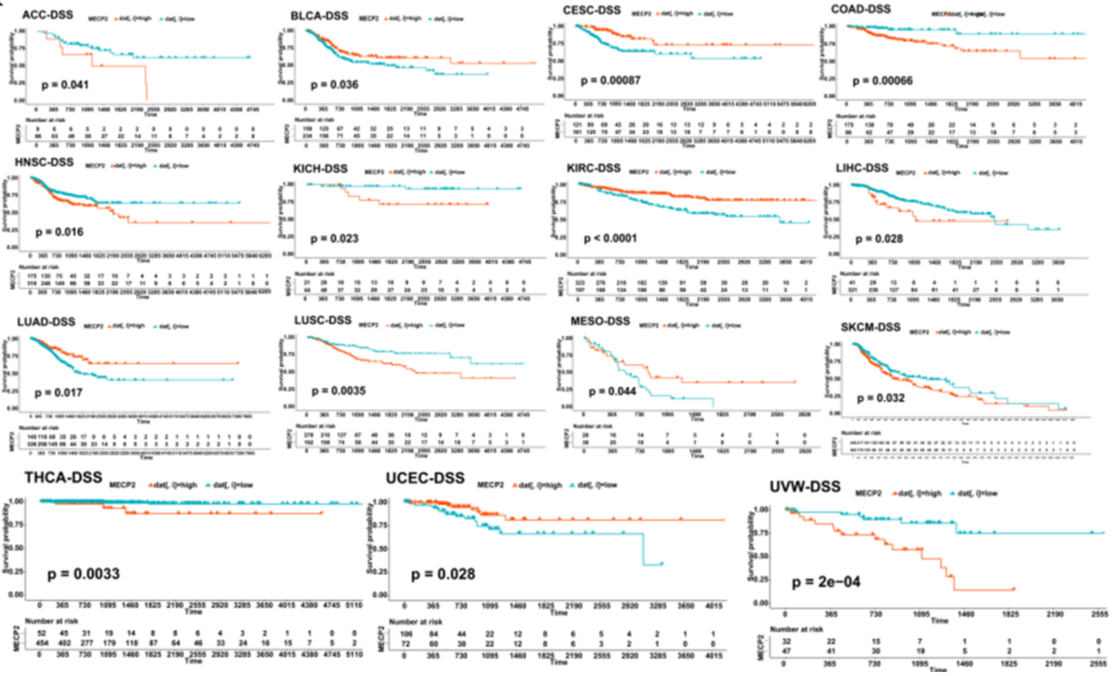
ACC-DSS
p = 0.041
BLCA-DSS
p = 0.036
CESC-DSS
p = 0.00087
COAD-DSS
p = 0.00066
HNSC-DSS
p = 0.016
KICH-DSS
p = 0.023
KIRC-DSS
p < 0.0001
LIHC-DSS
p = 0.028
LUAD-DSS
p = 0.017
LUSC-DSS
p = 0.0035
MESO-DSS
p = 0.044
SKCM-DSS
p = 0.032
THCA-DSS
p = 0.0033
UCEC-DSS
p = 0.028
UVW-DSS
p = 2e−04

B

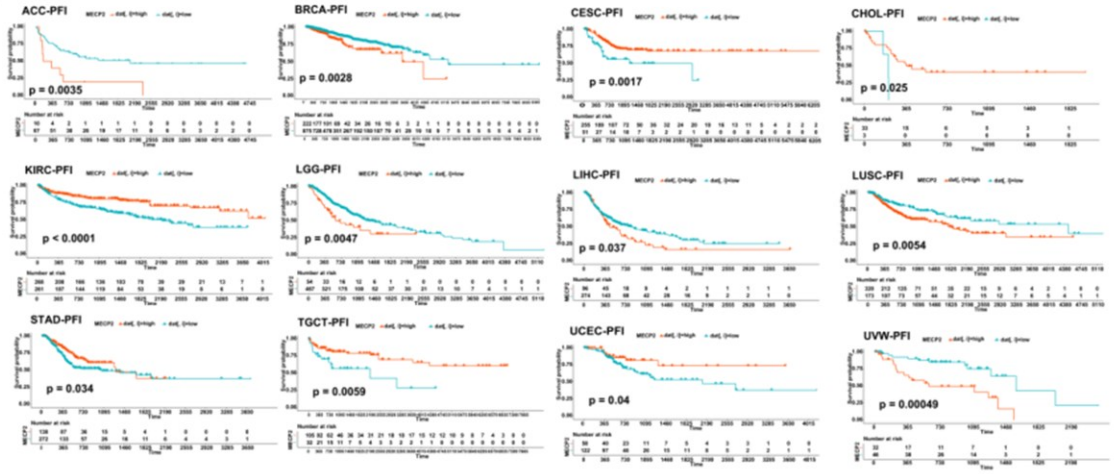
ACC-PFI
p = 0.0035
BRCA-PFI
p = 0.0028
CESC-PFI
p = 0.0017
CHOL-PFI
p = 0.025
KIRC-PFI
p < 0.0001
LGG-PFI
p = 0.0047
LIHC-PFI
p = 0.037
LUSC-PFI
p = 0.0054
STAD-PFI
p = 0.034
TGCT-PFI
p = 0.0059
UCEC-PFI
p = 0.04
UVW-PFI
p = 0.00049

C

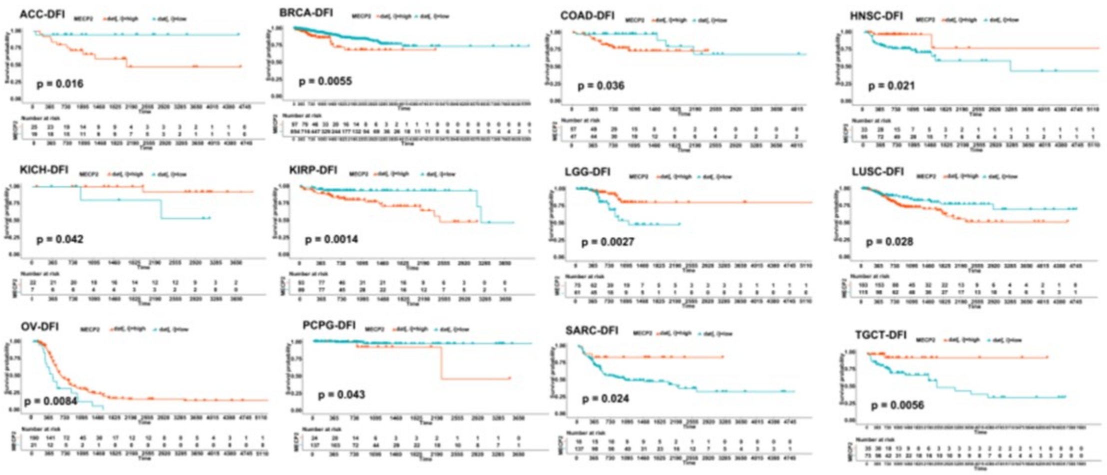

Figure S3. The correlation between MeCP2 expression and survival prognosis of pan-cancer. **(A).** DSS analyses of MeCP2 in pan-cancer(P<0.05). **(B).** PFI analyses of MeCP2 in pan-cancer(P<0.05). **(C).** DFI analyses of MeCP2 in pan-cancer(P<0.05). DFI, disease free interval; PFI,progression-free interval; DSS,disease-specific survival.

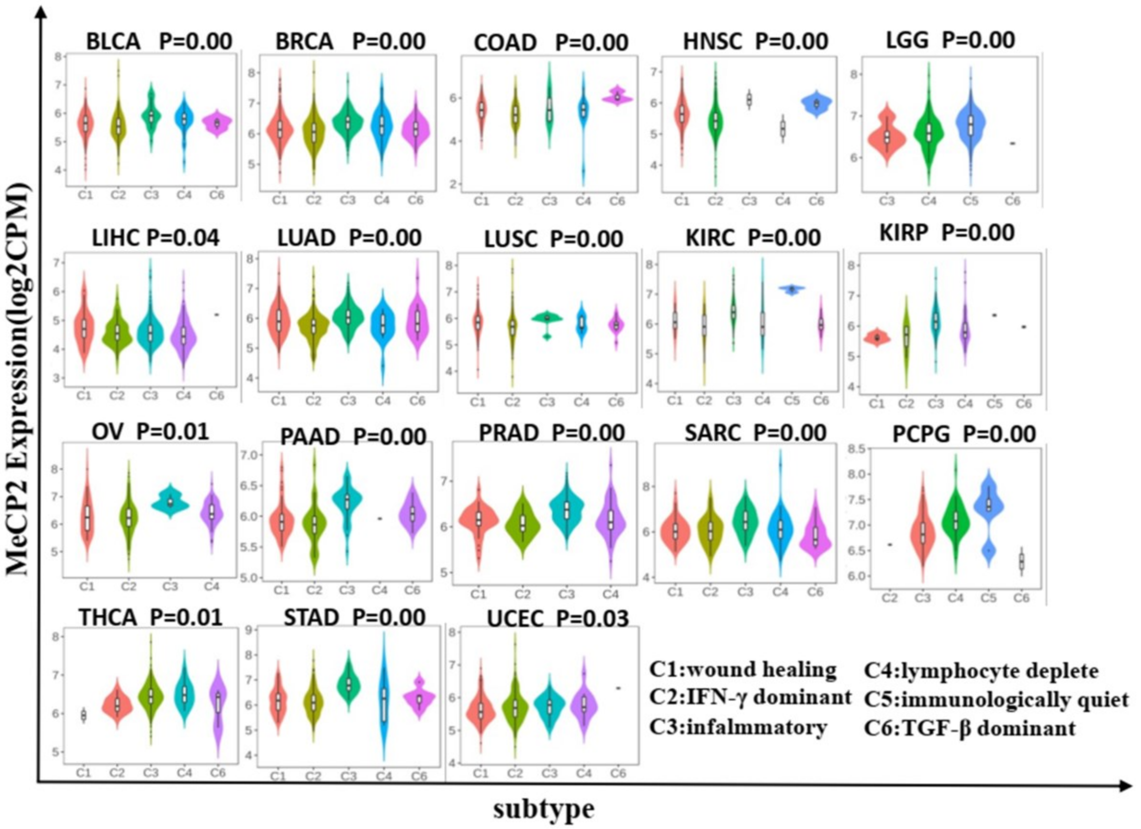

Figure S4. Correlation analysis between MePC2expression and immune subtype in cancers. A significant correlation between MeCP2 expression and immune subtypes in BLCA, BRCA, COAD, HNSC, LGG, LIHC, LUAD, LUSC, KIRC, KIRP, OV, PAAD, PRAD, SARC, PCPG, THCA, STAD and UCEC.(P<0.05)

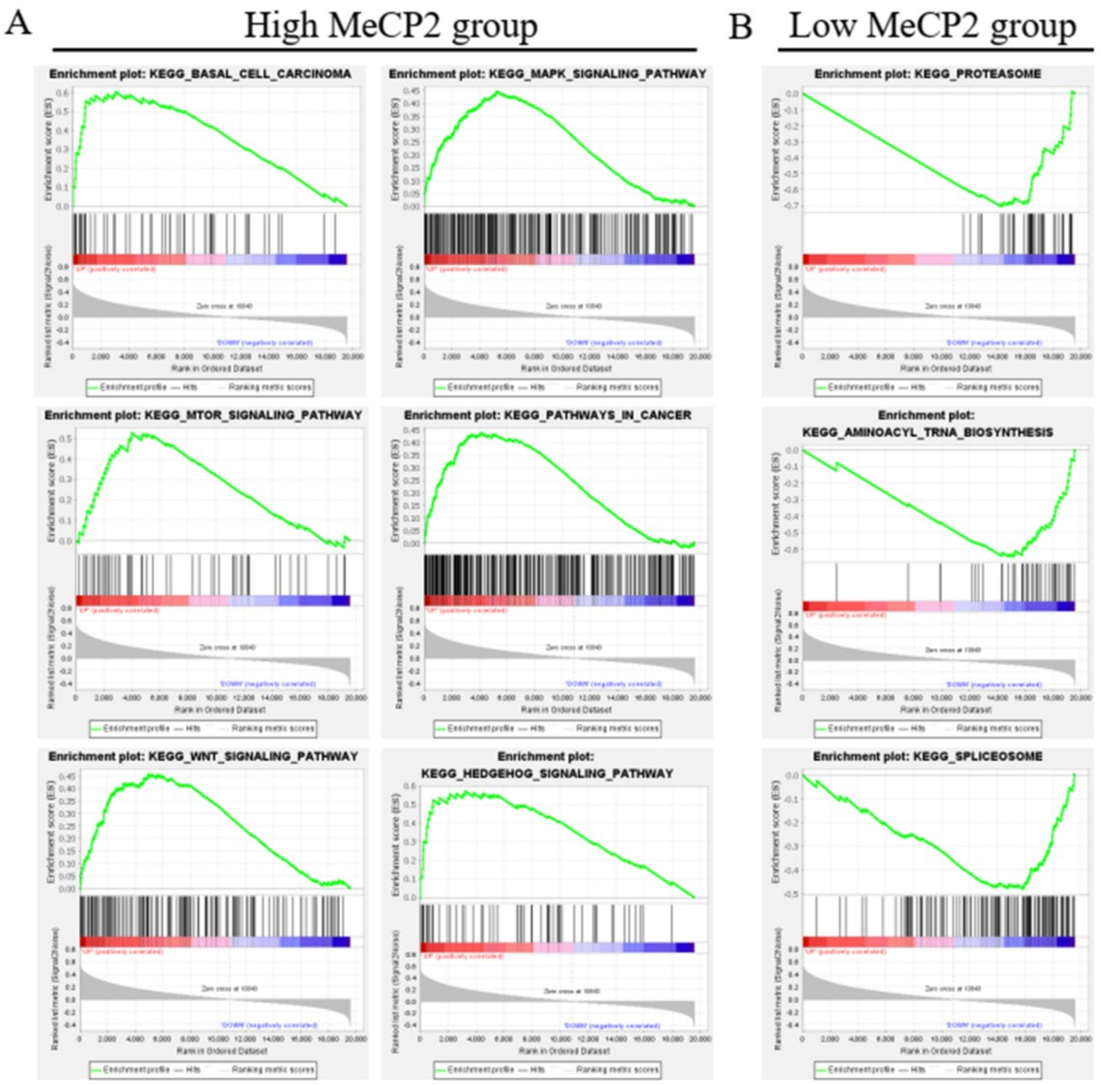

Figure S5. GSEA of MeCP2 in STAD.**(A).** The pathways were enriched in the high MeCP2 group. **(B).** The pathways were enriched in the low MeCP2 group. GSEA, Gene set enrichment analysis.

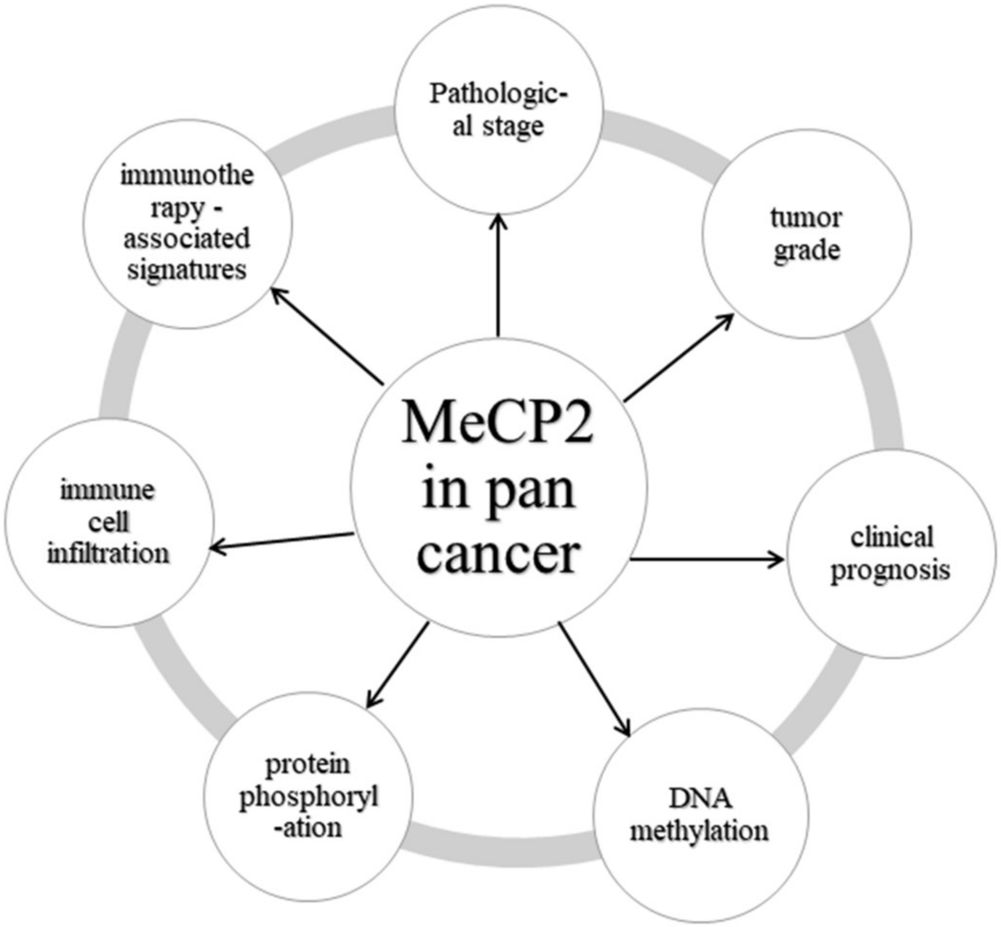

Figure S6. The concluding diagram of MeCP2 in pan-cancer.
